# Neonatal Septicemia in Nepal: Early-Onset versus Late-Onset

**DOI:** 10.1155/2015/379806

**Published:** 2015-11-16

**Authors:** Shamshul Ansari, Hari Prasad Nepal, Rajendra Gautam, Sony Shrestha, Puja Neopane, Moti Lal Chapagain

**Affiliations:** Department of Microbiology, Chitwan Medical College, Bharatpur, Chitwan, Nepal

## Abstract

*Introduction.* Neonatal septicemia is defined as infection in the first 28 days of life. Early-onset neonatal septicemia and late-onset neonatal septicemia are defined as illnesses appearing from birth to three days and from four to twenty-eight days postnatally, respectively.* Methods.* In this cross-sectional study, blood samples from the suspected infants were collected and processed in the bacteriology laboratory. The growth was identified by standard microbiological protocol and the antibiotic sensitivity testing was carried out by modified Kirby-Bauer disk diffusion method.* Results.* Among total suspected cases, the septicemia was confirmed in 116 (12.6%) neonates. Early-onset septicemia (EOS) was observed in 82 infants and late-onset septicemia (LOS) in 34 infants. Coagulase-negative staphylococcus (CoNS) (46.6%) was the predominant Gram-positive organism isolated from EOS as well as from LOS cases followed by* Staphylococcus aureus* (14.6%).* Acinetobacter* species (9.5%) was the predominant Gram-negative organism followed by* Klebsiella pneumoniae* (7.7%).* Conclusions.* The result of our study reveals that the CoNS,* Staphylococcus aureus*,* Acinetobacter* spp., and* Klebsiella pneumoniae* are the most common etiological agents of neonatal septicemia. In particular, since rate of CoNS causing sepsis is alarming, prompting concern to curb the excess burden of CoNS infection is necessary.

## 1. Introduction

Septicemia in neonates refers to generalized bacterial infection documented by a positive blood culture in the first 4 weeks of life [1]. Septicemia in neonates can lead to sepsis that is a clinical syndrome characterized by systemic signs of infection and accompanied by bacteremia [2]. Sepsis occurring in the first 72 hours of life is defined as early-onset sepsis (EOS) and that occurring beyond 72 hours as late-onset sepsis (LOS) [3].

Despite advances in health care, neonatal sepsis, and especially that caused by Gram-negative rod bacteria, is a significant cause of morbidity and mortality among neonates [4]. An increase in sepsis caused by Gram-negative organisms has been reported in recent years from Nepal [5, 6]. Neonatal sepsis caused by Gram-negative microorganisms is responsible for 18%–78% of all neonatal sepsis [7–11]. In the developing world,* Escherichia coli* (*E. coli*),* Klebsiella* species, and* Staphylococcus aureus* (*S. aureus*) are the most common pathogens of EOS, whereas* S. aureus*,* Streptococcus pneumoniae*, and* Streptococcus pyogenes* are the most commonly reported organisms in LOS [12, 13]. Moreover, the causative organisms of EOS and LOS sepsis are similar especially in hospital setting in developing country [14].

Microorganisms implicated in neonatal septicemia have developed increased drug resistance to commonly used antibiotics and thus making treatment extremely difficult [15]. Thus, the knowledge of both the common pathogens causing septicemia in neonates and their antimicrobial susceptibility is essential in order to select appropriate antimicrobial treatment. Moreover, antimicrobial susceptibility patterns of pathogens vary geographically and are temporally dependent on local pathogens and patterns of antibiotic used. Hence, the present study was conducted to document the bacteriological profile of neonatal septicemia and their antibiotic susceptibility profile for planning strategy for the management of neonatal septicemia.

## 2. Methods

A cross-sectional study was carried out from January 2012 to December 2013 at Chitwan Medical College Teaching Hospital (a 600-bed hospital) which is located at Bharatpur, Chitwan District of Nepal.

### 2.1. Study Population

A total of 918 febrile subjects aged up to 28 days with clinical features such as respiratory distress, poor feeding, lethargy, abdominal distension, apnea, irritability, vomiting, convulsions, and cyanosis suggestive of septicemia were enrolled in this study.

### 2.2. Sample Collection

One milliliter (mL) of blood samples were collected aseptically by clinicians or trained nurse using sterile syringe and needle by venipuncture and immediately the blood samples were carefully transferred into 9 mL of Brain Heart Infusion (BHI) broth and labeled with the patient's name, age/sex, identification number, date, and time of collection.

### 2.3. Bacteriological Processing

The BHI broth inoculated with blood sample was transported to the laboratory and incubated at 37°C in aerobic condition. Subcultures were made into sheep blood agar, chocolate agar, and MacConkey agar after overnight of aerobic incubation. Blood agar and MacConkey agar plates were incubated overnight at 37°C in aerobic atmosphere while chocolate agar plates were incubated overnight at 37°C in 5% CO_2_ atmosphere. Thereafter, culture bottles were observed for turbidity for up to 10 days. Final blind subcultures were done before reporting the sample negative. Growth obtained was identified by standard methods [16]. A purity plate was employed to ensure that the inoculum used for the biochemical tests was pure.

### 2.4. Antibiotic Susceptibility Testing

All the isolates grown were subjected to antibiotic susceptibility testing by modified Kirby-Bauer disk diffusion method in compliance with Clinical and Laboratory Standards Institute (CLSI) guidelines using Mueller-Hinton agar standard media. The inhibition zone standards for antimicrobial susceptibility were considered from tables for interpretative zone diameters of CLSI [17].

Antibiotic disks (HiMedia Laboratories, Pvt. Limited, India) used were oxacillin (1 *μ*g), erythromycin (15 *μ*g), clindamycin (2 *μ*g), vancomycin (30 *μ*g), teicoplanin (30 *μ*g), penicillin (10 U), cephalexin (30 *μ*g), cotrimoxazole (25 *μ*g), gentamicin (10 *μ*g), amikacin (30 *μ*g), ofloxacin (5 *μ*g), cefixime (5 *μ*g), cefotaxime (30 *μ*g), ceftazidime (30 *μ*g), piperacillin (100 *μ*g), piperacillin-tazobactam (100/10 *μ*g), carbenicillin (100 *μ*g), and ampicillin (10 *μ*g).

*Staphylococcus aureus* ATCC 25923 and* Escherichia coli* 25922 were used as a control organisms for antibiotic sensitivity testing.

### 2.5. Ethical Aspects

This study was approved by the Institutional Review Committee of Chitwan Medical College, Bharatpur, Nepal. Informed consent was obtained from the guardians of participating infants before collecting the specimens.

## 3. Results

### 3.1. Gender-Wise Distribution of Cases

Of total 918 enrolled neonates, 61.4% were males and 38.6% were females with male to female ratio of 1.59 : 1. Among total enrolled cases, septicemia was confirmed in 116 (12.6%) neonates among which EOS was found in 82 (70.7%) neonates while LOS was found in 34 (29.3%) neonates (Table 1).

### 3.2. Isolates Distribution

Among a total of 116 bacterial isolates recovered, 74 (63.8%) were Gram-positive isolates and 42 (36.2%) were Gram-negative isolates. Of total positive cases, CoNS were recovered from nearly half of the cases (46.6%) followed by* S. aureus* (14.6%),* Acinetobacter* spp. (9.5%), and* Klebsiella pneumoniae* (7.7%) whereas viridans streptococci was recovered from a single case.* E. coli* and* Proteus mirabilis* were recovered from LOS cases but not from EOS cases while* Enterococcus* spp., viridans streptococci, and* Burkholderia* spp. were recovered from EOS case but not from LOS cases (Table 2).

### 3.3. Antibiotic Resistance Characteristics of Isolates Recovered from EOS Septicemia

Vancomycin and teicoplanin showed 100% efficacy against Gram-positive isolates. Most of the Gram-positive isolates were resistant to penicillin, erythromycin, and gentamicin whereas amikacin showed a promising efficacy among tested antibiotics. Among Gram-negative isolates, nearly all the isolates were resistant to ampicillin and most of the isolates were resistant to cefixime and cefotaxime while amikacin was found to be most effective among tested antibiotics (Table 3).

### 3.4. Antibiotic Resistance Characteristics of Isolates Recovered from LOS

Of isolates recovered from LOS cases, most of the Gram-positive isolates were resistant to erythromycin, penicillin, and cephalexin while vancomycin and teicoplanin showed 100% efficacy and amikacin showed better efficacy among all the antibiotics tested. Among Gram-negative isolates, nearly all the isolates were resistant to ampicillin whereas nearly all* Citrobacter* spp. and* Proteus mirabilis* were susceptible to most of the antibiotic tested. Nearly all isolates of* Enterobacter* spp., up to 50%* E. coli* isolates, and 33.3% of the* Klebsiella* spp. were resistant to most of the antibiotic tested as elaborated in Table 4.

## 4. Discussions

Sepsis remains one of the most important causes of morbidity and mortality in the newborn despite considerable progress in hygiene, introduction of new antimicrobial agents, and advanced measures for early diagnosis and treatment [18, 19].

Of the infants born at a tertiary care center located at Bharatpur of central Nepal between January 2012 and December 2013, the septicemia was suspected in 918 infants. Among them 61.4% infants were males and 38.6% were female infants. Similar rate of suspected septicemia in male and female infants was also reported by Karambin and Zarkesh from Iran [20] and Al-Shamahy et al. from Yemen [21].

In our setting, the burden of septicemia among total suspected cases was confirmed in 116 infants by positive blood culture growth giving a prevalence rate of 12.6% which is a lower rate than previously reported by Khanal et al. [5] from eastern Nepal. The lower rate observed in our study may be due to the multiple changes that have occurred with increasing awareness of prevention of sepsis. These changes include prevention of preterm labor, earlier and more aggressive enteral feeding and early discontinuation of vascular catheters, shorter duration of invasive ventilation because of surfactant use, better hand hygiene practices, and better protocols for handling vascular lines [14]. However, similar rate of positivity was also detected by Dagnew et al. from Ethiopia [22], Mhada et al. from Tanzania [23], Karambin and Zarkesh from Iran [20], and Mutlu et al. from Turkey [24].

The microorganisms responsible for neonatal sepsis have changed over time, and they vary markedly from region to region. Prematurity, frequent use of catheters, use of total parenteral nutrition, and frequent antibiotic resistance were all reported as causes of change in the etiology of neonatal sepsis [25].

The impacts of specific etiologic agents on blood stream infected patient outcome are tremendous; blood stream infection increases the mortality rate, prolongs patient stay in an intensive care unit and in the hospital, and leads to increased health care costs [26, 27]. Among the culture proven cases, bacterial septicemia was observed mostly in male neonates (69.8%) in the present study whereas it was confirmed to be 30.2% in female neonates. Similar finding of septicemia in male and female neonates was also detected by Karambin and Zarkesh [20] and Naher and Khamael from Iraq [28].

Since etiologic agents in neonatal EOS and sometimes LOS are often acquired from mother's genital tract, vaginal cultures in all pregnant women as a screening program and appropriate treatment of positive cases before delivery should be emphasized. Among total septicemia cases EOS was found in 82 (70.7%) neonates and LOS was found in 34 (29.3%) neonates. The result indicated that the incidence of EOS septicemia was more common than LOS which is consistent with other reports from Nepal [6], Iran [29, 30], Iraq [28], Bangladesh [31], and Yemen [21].

Although Gram-positive organisms are the most common causes of nosocomial blood stream infections, Gram-negative bacteremia carries higher risks of severe sepsis, septic shock, and death. Sundaram et al. reported a neonatal mortality rate due to Gram-negative sepsis of 34% to 55% [14]. Even in the present study, Gram-positive organism constituted the major group of isolates accounting for 63.8%. The higher proportion of Gram-positive organism in this study corroborates with 74% reported by Khanal et al. from Nepal [5], 69% reported by Dagnew et al. from Ethiopia [22], and 68% reported by Mutlu et al. from Turkey [24].

Early-onset neonatal sepsis is caused by microorganisms acquired from the mother before or during birth (vertically transmitted and perinatally acquired); thus, microorganisms from the maternal genital tract may play an important role in early infection [32]. Among Gram-positive group of organisms CoNS was the most common cause of both EOS and LOS accounting for nearly half of the cases (46.6%) followed by* S. aureus* (14.6%). Similar rates of CoNS and* S. aureus* isolates were also reported by Dagnew et al. from Ethiopia [22], Ozkan et al. from Turkey [25], Karambin and Zarkesh [20], and Ghotaslou et al. [33] from Iran. The interpretation of the CoNS to be a cause of septicemia is a major concern for clinicians and clinical microbiology laboratories. The observation of sepsis symptoms and the number of positive blood cultures usually confirms the decision for therapy. However, in this study, the criteria of multiple blood culture were not applied because we could not go for multiple blood sample collection in early age patients. Among Gram-negative organisms,* Acinetobacter* spp. (9.5%) were the most common organism isolated from EOS cases while* Enterobacter* spp. were the predominant organism from LOS cases.

Neonatal sepsis is a life threatening emergency and thus any delay in treatment may cause death. The knowledge of the etiological organisms as well as their antimicrobial sensitivity profile is necessary for commencement of antibiotic therapy empirically while awaiting blood culture results. The initial empiric antibiotic therapy must therefore be a combination of drugs to cover the prevalent bacterial organisms in that locality.

The present study has shown the sensitivity pattern of the common pathogens isolated from EOS as well as LOS to commonly used antibiotics. Aminoglycosides (gentamicin and amikacin) and quinolones (ofloxacin) were observed to be the most effective antimicrobial agents against both Gram-positive and Gram-negative organisms while *β*-lactam antibiotics (ampicillin, penicillin, and cephalosporins) were observed as the least effective ones against them in our hospital. Similar pattern of susceptibility was also reported from Nepal [34], India [35], Tureky [25], and Pakistan [36].

Vancomycin and teicoplanin remained the most effective antibiotics against all the Gram-positive isolates from EOS as well as LOS cases; not a single case of resistant isolate was found against vancomycin and teicoplanin. Similarly, vancomycin was also found as the most effective antibiotic in a study by Komolafe and Adegoke from Nigeria [37] and Desai and Malek from India [35]. All of the Gram-positive organisms isolated from both EOS and LOS cases were also found to be susceptible to vancomycin by Ozkan et al. from Turky [25].

In EOS, minority of Gram-positive and Gram-negative isolates were sensitive to commonly tested antibiotics in the current study. Most of the Gram-positive isolates were resistant to erythromycin and gentamicin. Similar proportion of resistance rate in Gram-positive organisms to common antibiotics was also reported by Gheibi et al. from Iran [30]. Among Gram-negative isolates, nearly all the isolates were resistant to amoxicillin, the result being similar to that reported by Mhada et al. from Tanzania [23]. Relatively, amikacin was found to be fairly effective among the tested antibiotics. Effectiveness of amikacin was also highlighted by the literatures from Tanzania [23] and Turkey [24].

The antimicrobial sensitivity pattern differs in different studies as well as at different times in the same hospital. This is because of emergence of resistant strains as a result of indiscriminate use of antibiotics. In LOS, most of the Gram-positive isolates were resistant to erythromycin, penicillin, and cephalexin while amikacin was found to be effective regimen among the tested antibiotics. Among Gram-negative isolates, nearly all the isolates were resistant to ampicillin and the* Enterobacter* spp., and the predominant Gram-negative organism was resistant to most of the antibiotics tested. Nearly all Gram-negative isolates were also reported to be resistant to ampicillin by Desai and Malek from India [35]. Most of the* Enterobacter* spp. isolates tested by Karambin and Zarkesh in Iran also exhibited resistance to several antibiotics tested [20]. High resistance noted in this study may be primarily attributed to excessive and irrational use of these antibiotics at primary health care facilities and private clinics from which neonates are referred to our center.

## 5. Conclusions

This research study identified CoNS,* S. aureus*,* Acinetobacter* spp., and* Klebsiella pneumoniae* as the predominant etiological agents of bloodstream infection among neonates at CMCTH. Effective prophylactic measures, prompt and accurate diagnoses, and subsequent administration of targeted therapy are vital to curb the excessive burden of the disease. An alarmingly high degree of antibiotic resistance observed calls for an urgent evaluation and development of antibiotic policies and protocols for neonatal sepsis. Future epidemiological and clinical studies are also needed to monitor changes in the microorganisms causing neonatal sepsis.

## Figures and Tables

**Table 1:** Sex-wise distribution of total and septicemic cases.

Sex	Total cases (%)	Positive cases (%)		
		Early-onset	Late-onset	Total cases (%)
Male	564 (61.4)	59 (72)	22 (64.7)	81 (69.8)
Female	354 (38.6)	23 (28)	12 (35.3)	35 (30.2)

Total	**918 (100)**	**82 (100)**	**34 (100)**	**116 (100)**

**Table 2:** Distribution of isolated organisms.

Organism isolated	Frequency		Total (%)
	Early-onset (EOS)	Late-onset (LOS)	
Gram-positive organisms	**60**	**14**	**74 (63.8)**
*S. aureus*	12	5	17 (14.6)
CoNS	45	9	54 (46.6)
*Enterococcus *spp.	2	0	2 (1.7)
*Viridans streptococci*	1	0	1 (0.85)
Gram-negative organisms	**22**	**20**	**42 (36.2)**
*Acinetobacter *spp.	7	4	11 (9.5)
*Pseudomonas aeruginosa*	5	1	6 (5.2)
*Citrobacter *spp.	1	1	2 (1.7)
*E. coli*	0	4	4 (3.4)
*Enterobacter *spp.	1	5	6 (5.2)
*Klebsiella pneumoniae*	6	3	9 (7.7)
*Burkholderia *spp.	2	0	2 (1.7)
*Proteus mirabilis*	0	2	2 (1.7)

Total	**82**	**34**	**116 (100)**

**Table 3:** Resistance rates of isolates recovered from early-onset cases.

Isolated organisms (*N*)	Resistance rates to different antibiotic tested															
	OX	E	CD	P	CFX	COT	G	AK	OF	CFM	CTX	CAZ	PI	PIT	CAR	AMP
*S. aureus* (12)	33.3	83.3	50	91.7	66.7	75	58.3	25	41.6	—	—	—	—	—	—	—
CoNS (45)	15.5	44.4	31.1	60	62.2	57.8	48.9	11	29	—	—	—	—	—	—	—
*Enterococcus *spp. (2)	0	100	0	100	50	—	100	50	50	—	—	—	—	—	—	—
*Viridans streptococci* (1)	—	0	0	100	—	0	0	0	0	—	—	—	—	—	—	—
*Acinetobacter *spp. (7)	—	—	—	—	—	14.3	28.6	14.3	28.6	42.8	28.6	57.1	57.1	42.8	—	85.6
*Pseudomonas aeruginosa* (5)	—	—	—	—	—	—	20	0	20	80	60	20	40	20	20	—
*Citrobacter *spp. (1)	—	—	—	—	—	0	0	0	100	0	0	0	—	—	—	100
*Enterobacter *spp. (1)	—	—	—	—	—	100	0	0	0	100	100	100	0	0	—	100
*Klebsiella *spp. (6)	—	—	—	—	—	50	50	16.7	16.7	66.7	50	33.3	33.3	16.7	—	100
*Burkholderia *spp. (2)	—	—	—	—	—	50	50	0	50	100	50	0	0	0	—	100

OX: oxacillin, E: erythromycin, CD: clindamycin, P: penicillin, CFX: cephalexin, COT: cotrimoxazole, G: gentamicin, AK: amikacin, OF: ofloxacin, CFM: cefixime, CTX: cefotaxim, CAZ: ceftazidime, PI: piperacillin, PIT: piperacillin-tazobactam, CAR: carbenicillin, and AMP: ampicillin.

—: not tested.

**Table 4:** Resistant rates of isolates recovered from late-onset septicemia cases.

Isolated organisms (*N*)	Resistance rates to different antibiotic tested															
	OX	E	CD	P	CFX	COT	G	AK	OF	CFM	CTX	CAZ	PI	PIT	CAR	AMP
*S. aureus* (5)	40	60	40	80	80	40	40	0	20	—	—	—	—	—	—	—
CoNS (9)	44.4	66.7	22.2	89	77.8	55.5	22.2	11.1	22.2	—	—	—	—	—	—	—
*Acinetobacter *spp. (4)	—	—	—	—	—	75	0	0	0	75	75	75	25	0	—	100
*Pseudomonas aeruginosa* (1)	—	—	—	—	—	—	100	0	0	100	100	0	100	100	0	—
*Citrobacter *spp. (1)	—	—	—	—	—	0	0	0	0	0	0	0	0	0	—	100
*E. coli* (4)	—	—	—	—	—	50	50	25	25	25	25	25	0	0	—	75
*Enterobacter *spp. (5)	—	—	—	—	—	100	100	80	100	100	100	80	80	60	—	100
*Klebsiella *spp. (3)	—	—	—	—	—	33.3	33.3	33.3	33.3	33.3	33.3	33.3	—	—	—	100
*Proteus mirabilis* (2)	—	—	—	—	—	0	50	0	0	0	0	0	0	0	—	100

OX: oxacillin, E: erythromycin, CD: clindamycin, P: penicillin, CFX: cephalexin, COT: cotrimoxazole, G: gentamicin, AK: amikacin, OF: ofloxacin, CFM: cefixime, CTX: cefotaxim, CAZ: ceftazidime, PI: piperacillin, PIT: piperacillin-tazobactam, CAR: carbenicillin, and AMP: ampicillin.

—: not tested.
